# Awareness of Gingival Recession and Its Causes and Consequences Among Adults in Saudi Arabia

**DOI:** 10.3390/dj13110501

**Published:** 2025-10-28

**Authors:** Marwa Madi, Eman Aljoghaiman, Shahad T. Alameer, Mohammed Albander, Muntathir Alahmed, Mujtaba Almuallim, Ahmed Elakel, Maha Abdelsalam

**Affiliations:** 1 Department of Preventive Dental Sciences, College of Dentistry, Imam Abdulrahman Bin Faisal University, P.O. Box 1982, Dammam 31441, Saudi Arabia; ealjoghaiman@iau.edu.sa (E.A.); amelakel@iau.edu.sa (A.E.); 2 College of Dentistry, Imam Abdulrahman Bin Faisal University, P.O. Box 1982, Dammam 31441, Saudi Arabia; 2180000445@iau.edu.sa (S.T.A.); 2180002380@iau.edu.sa (M.A.); 2180002312@iau.edu.sa (M.A.); 2180003989@iau.edu.sa (M.A.); 3 Department of Biomedical Dental Sciences, College of Dentistry, Imam Abdulrahman Bin Faisal University, P.O. Box 1982, Dammam 31441, Saudi Arabia; mmabdelsalam@iau.edu.sa

**Keywords:** awareness, adverse effects, causality, dentin sensitivity, gingival recession

## Abstract

**Background/Objectives**: Gingival recession (GR), characterized by the apical displacement of the gingival margin leading to root exposure, risk of root caries, dentine hypersensitivity (DH), and plaque accumulation. This study aimed to evaluate the awareness, causes, and consequences of gingival recession among adults in Saudi Arabia. **Methods**: A cross-sectional self-reported survey was conducted from September 2023 to December 2024, involving 619 participants (51.53% male). Participants were recruited through dental clinics, community centers, and online platforms across multiple regions in Saudi Arabia to ensure diverse demographic and socioeconomic representation. A validated 27-question survey collected data on demographics, oral hygiene practices, and GR awareness and related factors. Statistical analysis was performed using SAS 9.4, with significance set at *p* < 0.05. **Results:** The prevalence of GR was 26.66%, dental plaque was the most frequently reported causing factor, followed by medical conditions (45.4%). Aesthetic concerns were the most recognized consequence (78.4%) followed by periodontitis and tooth mobility and tooth loss (58.5%). Medical disease (Odds Ratio OR = 2.149, *p* < 0.0001), trauma (OR = 1.515, *p* = 0.0078), and rough brushing (OR = 1.431, *p* = 0.0233) were identified as significant risk factors for gingival recession. The association between gingival recession (GR) and its perceived consequences was generally not statistically significant. However, a significant relationship was observed with dental caries (*p* = 0.0472). **Conclusions:** Gingival recession awareness among Saudi adults was influenced by age, gender, smoking, and oral hygiene factors. The findings emphasize the importance of raising awareness and promoting preventive strategies targeting modifiable risk factors to reduce GR prevalence and clinical impact.

## 1. Introduction

Gingival recession (GR) is defined as the displacement of the gingival margin apical to the cementoenamel junction (CEJ), resulting in root exposure. Exposed root surfaces are prone to abrasion, plaque accumulation, and calculus formation, increasing the risk of root caries and dentine hypersensitivity [1]. The prevalence and severity of GR increase with age, affecting at least 40% of young adults and up to 88% of elderly patients, with at least one site showing 1 mm or more of recession [2].

A new classification system for GR, introduced in the 2018 World Workshop Classification System, categorizes GR into three types; RT1, RT2, and RT3 based on inter-dental clinical attachment loss [3]. This system has facilitated more precise diagnosis and treatment planning. Numerous surgical techniques have been developed to address mid-buccal GR and restore esthetic and functional conditions [3].

Epidemiological studies reveal significant variations in GR prevalence across demographics [4,5]. Research on young Greek adults found a slightly higher prevalence in males (29 out of 51) compared to females (27 out of 53), with 56 out of 104 individuals exhibiting at least one tooth with a denuded root surface >1.0 mm. Among 2912 examined teeth, 124 (4.3%) demonstrated GR [4]. Similarly, a study of young European adults reported that all of the 349 participants had GR, with 58.4% exhibiting a maximum recession depth of 1–3 mm and 41.6% showing 4–8 mm recession [5].

GR is a multifactorial condition predominantly affecting adults. If left untreated, it can progress from initial symptoms such as tooth sensitivity and esthetic concerns to severe complications, including tooth loss [1]. Etiological studies highlight improper toothbrushing techniques as a significant contributing factor (42.71%), while 39.7% of dental practitioners identify traumatic occlusion as a risk factor. Additionally, 29.65% GR were attributed to accidental toothbrush trauma [1,6,7].

Despite its high prevalence, patient awareness of GR remains limited. Shetty et al. reported that 83.3% of their study population lacked awareness of GR, with only 9.4% were informed about its various causes [8]. Similarly, Neire et al. found that out of 120 enrolled patients, 96 exhibited 783 gingival recessions, of which 565 (72.2%) went unnoticed by the patients and only 218 recessions were perceived [9]. These findings emphasize significant gaps in patient awareness and understanding of GR, particularly its causes, risk factors, and consequences. In Saudi Arabia, previous study [10] found that 73% of Southwestern Saudi adolescent males exhibited gingival recession. The study also identified factors such as neglecting oral hygiene and the use of Miswak as significant contributors to gingival recession in this population. Another study [11] found that more than half of Saudi adults in the studied 748 persons reported dentine hypersensitivity, with substantial quality of life implications. 

Despite GR prevalence, its awareness, causes, and consequences remain limited, which may compromise timely prevention and management. Therefore, the aim of this study was to investigate GR awareness, associated factors and consequences among the Saudi adult population, which could provide perspective data that can guide directed educational and preventive approaches.

The null hypothesis of this study is that there is no association between demographic factors, oral hygiene practices, and the awareness, causes, or consequences of gingival recession among adults in Saudi Arabia.

## 2. Materials and Methods

This study employed a cross-sectional design using a self-reported questionnaire to assess the awareness, causes, and consequences of gingival recession (GR) among adults in Saudi Arabia. The study was approved by the Institutional Review Board (IRB) of Imam Abdulrahman Bin Faisal University (2022-02-372. 11 October 2022). Participants were informed about the study’s purpose, and a written consent was obtained before participation. Data was anonymized to ensure confidentiality. The questionnaire was designed to collect data on demographic characteristics, oral hygiene practices, and GR-related factors. The study was conducted from September 2023 to December 2024. Based on confidence interval 5 and a confidence level of 95%, a minimum sample size of 384 participants was needed.

Participants:

The study included participants from both genders from diverse demographic backgrounds in Saudi Arabia. Participants were recruited through multiple sources to ensure a representative and diverse sample. Recruitment was conducted via dental clinics, community centers, and online platforms (including social media). This multi-source method allowed inclusion of individuals from different geographic regions across Saudi Arabia. By combining in-person and online recruitment, we minimized potential bias associated with single-site sampling and ensured coverage of varying socioeconomic and demographic backgrounds. Inclusion criteria were participants of more than 18 years, and their ability and willingness to complete the questionnaire in Arabic or English. Exclusion criteria included individuals with cognitive impairments or those unwilling to provide informed consent.

Questionnaire Development and Validation:

To ensure methodological rigor, the 27-item questionnaire was developed based on previous validated survey [12] and reviewed by a panel of oral health experts. A pilot test was conducted on 22 participants to confirm clarity and reliability. The survey was distributed through dental clinics, community centers, and online platforms, with duplicate responses prevented by restricting one entry per device. Questionnaires with more than 20% missing data were excluded from analysis. The study design and reporting followed the Consensus-Based Checklist for Reporting of Survey Studies (CROSS) guidelines.

The questionnaire consisted of closed-ended questions that were divided into four sections (Supplementary Material S1):

1. Demographic Information: Age, gender, marital status, educational level, and socioeconomic status.
2. Oral Hygiene and Dental Care Practices: medical condition, Smoking habits [13], dental clinic preferences, frequency of dental visits, brushing frequency, and brushing technique.
3. Gingival Recession and Oral Health Status: Number of missing teeth (excluding wisdom teeth), presence of dentine hypersensitivity (DH), number of teeth affected by DH, and awareness of DH treatments.
4. Knowledge of Gingival Recession:

This section assessed participants’ awareness of GR, the number of teeth affected by GR, and their knowledge of its causes, consequences, and treatments. Participants were asked about:

Perceived causes of GR (e.g., trauma, rough brushing, plaque accumulation, medical diseases), with response options on a 3-point Likert scale: Yes, Maybe, No.

Perceived consequences of GR (e.g., aesthetic concerns, tooth mobility, tooth loss, periodontitis, caries), with response options on a 3-point Likert scale: Leads to, I do not know, Irrelevant.

Knowledge of GR treatments, with response options: Yes or No.

The questionnaire was validated for content and clarity by a panel of three dental experts and piloted on a small sample (*n* = 30) to ensure comprehensibility and reliability. Cronbach’s alpha was calculated to assess internal consistency, with a value of 0.82, indicating good reliability.

Data Collection, Informed Consent and Confidentiality

Data was collected through self-administered questionnaires, distributed both online (via Google Forms) and in person at dental clinics and community centers. Participants were provided with a detailed explanation of the study’s purpose and assured of the confidentiality of their responses. Participants could withdraw at any point without consequences. Informed consent was obtained from all participants before they completed the questionnaire. The survey data collected was kept anonymous and confidential to ensure participant privacy. To prevent duplication, participants were permitted to complete the questionnaire only once. Additionally, all incomplete questionnaires were excluded from the statistical analysis to maintain data integrity and accuracy.

Statistical Analysis

Data were analyzed using SAS 9.4 (SAS Institute Inc., Cary, NC, USA). Correct answers in each section were defined, and internal consistency was assessed using Cronbach’s alpha. Descriptive statistics were used to summarize demographic characteristics, oral hygiene practices, gingival recession–related factors, and participants’ knowledge about gingival recession. Categorical variables were presented as frequencies and percentages. Associations between categorical variables were assessed using chi-square tests, and logistic regression analysis was employed to identify predictors of gingival recession. Multivariable logistic regression models were adjusted for potential confounders, including age, gender, socioeconomic status, and smoking habits. Interaction terms (e.g., gender × smoking, gender × socioeconomic status) were examined but were not retained in the final model if not statistically relevant. Adjusted odds ratios (OR) with 95% confidence intervals (CI) were reported.

## 3. Results

The study included 619 participants, with a slight majority being male (51.53%). The age distribution revealed that 39.58% of participants were aged 18–25 and 26.49% aged 26–35. Regarding marital status, 67.21% were married, and educational levels were 58% of participants holding a university degree. Most participants (56.38%) belonged to the low socioeconomic class (<7000 SR). Among the participants, 26.66% reported to have gingival recession (GR) with only 5.6–6.7% having recessions in 4 or more of their teeth, respectively. Among participants, 252 (40.7%) reported having DH, and 26.6% reported having GR. Most of the participants reported having GR (330 participants 53.3%) had no DH, meanwhile participants with more than 4 teeth affected by DH were only 11.15%.

A total of 85.46% of participants were non-smokers, and 32% of them brushed 1–2 times/day, while only 8% brushed 3 times and only 2% never brushed their teeth.

Among participants reported having GR, 53% brushed less frequently, compared to those who brushed 2 times/day, with only 24.38% reported having GR. Regarding the brushing technique, the random tooth brushing showed the highest prevalence of GR reports (31.58%) followed by the circular technique 26%. Most of our participants, 81.8%, do not know the treatment of DH; in addition, 73.6% of them did not report having GR. The participant characteristics are presented in Table 1.

**Figure 1:**
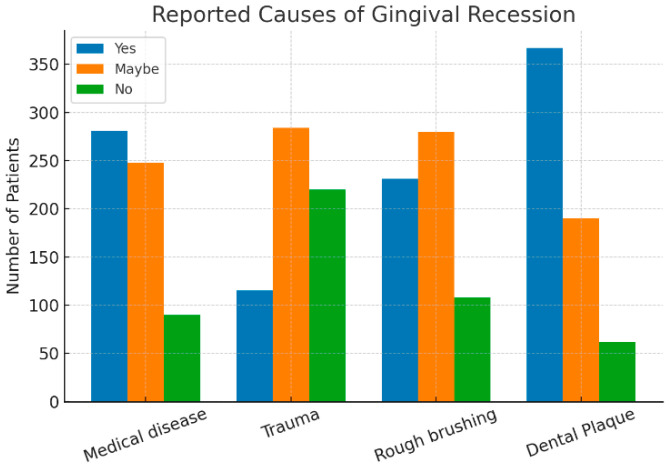
The reported causes of gingival recession.

The study analyzed the potential causes and consequences of gingival recession, including medical conditions, trauma, rough brushing, and plaque accumulation. Plaque was the most frequently reported factor, with 59.3% of participants identifying it as a cause. It was followed by medical conditions (45.4%), rough brushing (37.3%), and trauma (18.6%), Figure 1.

**Figure 2:**
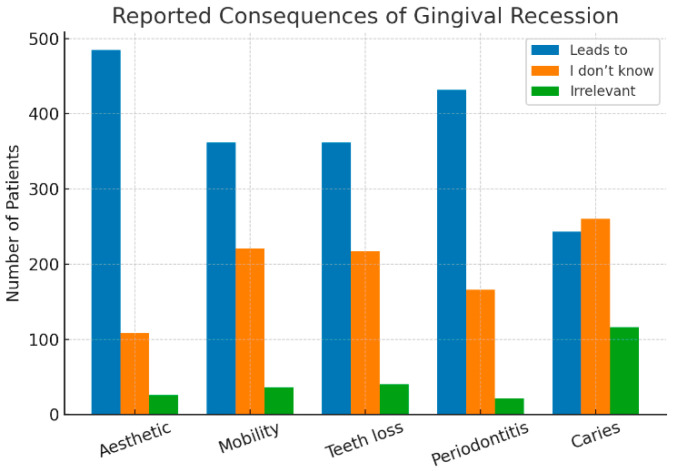
The reported consequences of gingival recession.

Participants were surveyed regarding their perception of GR consequences, including aesthetic concerns, mobility, tooth loss, periodontitis, and caries. Aesthetic concerns were the most recognized consequence, with 78.4% of participants acknowledging its impact. Periodontitis was identified as a consequence by 69.8%, followed by tooth mobility (58.5%) and tooth loss (58.5%). Caries was reported by 39.3% as a potential consequence, with 42% unsure of its association with GR. Periodontitis was identified as a consequence by 69.8%, followed by tooth mobility (58.5%) and tooth loss (58.5%). Caries was reported by 39.3% as a potential consequence, with 42% unsure of its association with GR, Figure 2.

Chi-square tests revealed significant associations between GR and several participant characteristics (Table 2). Prevalence of GR was higher in older age groups (*p* < 0.0001), in males (30.4% vs. 22.7%, *p* = 0.0295), among participants with higher socioeconomic status (*p* = 0.0006), and in smokers, especially heavy and former smokers (*p* = 0.0047). GR prevalence also increased with the number of missing teeth (*p* < 0.0001).

Brushing frequency and brushing technique were not significantly associated with GR (*p* = 0.126 and *p* = 0.1743, respectively). In contrast, dentin hypersensitivity and the number of hypersensitive teeth were strongly associated with GR (both *p* < 0.0001). Awareness of treatment for GR was not significantly related (*p* = 0.138).

**Table 2:** Bivariate chi-square analysis of participants’ characteristics and presence of gingival recession (GR).

	Presence of GR		
Age	Yes	No	*p*
20–29	16.33	83.67	0.0001 *
30–39	32.32	67.68	
40–49	35.77	64.23	
50–59	32.26	67.74	
>60	27.27	72.73	
Gender	Yes	No	*p*
Male	30.41	69.59	0.0295 *
Female	22.67	77.33	
Socioeconomic	Yes	No	*p*
<7000	20.63	79.37	0.0006 *
7000–20,000	34.22	65.78	
>20,000	35.56	64.44	
Smoking	Yes	No	*p*
Non	25.14	74.86	0.0047 *
Light	21.05	78.95	
Medium	27.91	72.09	
Heavy	64.29	35.71	
Former	50.00	50.00	
Missing teeth	Yes	No	*p*
0	19.44	80.56	<0.0001 *
1	21.82	78.18	
2	33.80	66.20	
3	37.21	62.79	
4	38.81	61.19	
>4	47.50	52.50	
Brushing frequency	Yes	No	*p*
3/day	30.00	70.00	0.126
2/day	24.38	75.62	
1/day	24.37	75.63	
Never	53.33	46.67	
Irregular	28.85	71.15	
Brushing technique	Yes	No	*p*
Horizontally	22.05	77.95	0.1743
Vertically	22.22	77.78	
Circularly	26.63	73.37	
Randomly	31.58	68.42	
Hypersensitivity	Yes	No	*p*
Yes	40.08	59.92	<0.0001 *
No	17.44	82.56	
Teeth affected by Hypersensitivity	Yes	No	*p*
0	16.67	83.33	<0.0001 *
1	22.73	77.27	
2	31.58	68.42	
3	40.91	59.09	
4	41.07	58.93	
>4	50.72	49.28	
Treatment of GR	Yes know	No know	*p*
Yes they have GR	32.17	67.83	0.1380
NO do not have GR	25.40	74.60	
Teeth affected by HS	Yes	No	*p*
0	1.59	98.41	<0.0001 *
1	61.29	38.71	
2	81.82	18.18	
3	96.43	3.57	
4	97.14	2.86	
>4	100.00	0.00	

* *p* < 0.05 showing significant difference. GR: gingival recession, HD: hypersensitivity.

Bivariate analysis of consequences (Table 3) showed no significant associations between GR and numbness, tooth mobility, tooth loss, or periodontitis (all *p* > 0.05). However, caries was significantly associated with GR (*p* = 0.0472), indicating that participants perceiving GR as a cause of caries had higher prevalence rates. This suggests that while most commonly assumed consequences of GR (such as mobility and periodontitis) were not independently linked in this sample, the link between GR and caries may be of clinical importance, Table 4.

Multivariable logistic regression confirmed that medical disease (OR = 2.15, 95% CI: 1.56–2.96), trauma (OR = 1.52, 95% CI: 1.12–2.06), and rough brushing (OR = 1.43, 95% CI: 1.05–1.95) were independent predictors of GR, whereas dental plaque showed a protective association (OR = 0.50, 95% CI: 0.35–0.71) (Table 4). None of the assessed consequences, including aesthetics, tooth mobility, tooth loss, periodontitis, or caries, were statistically significant predictors in the regression model.

**Table 4:** Multivariable logistic regression analysis of causes and consequences of gingival recession (GR).

Causes of GR	OR	95% CI	*p*
Medical Disease	2.149	1.561–2.957	<0.0001 *
Trauma	1.515	1.116–2.059	0.0078 *
Rough brushing	1.431	1.050–1.949	0.0233 *
Dental Plaque	0.501	0.352–0.714	0.0001 *
Consequences of GR	OR	95% CI	*p*
Anesthetic	0.897	0.579–1.390	0.6270
Mobility	1.001	0.671–1.495	0.9942
Tooth loss	1.262	0.860–1.852	0.2338
Periodontitis	0.850	0.557–1.298	0.4526
Caires	0.823	0.620–1.091	0.1757

* *p* < 0.05 showing significant difference.

**Figure 3:**
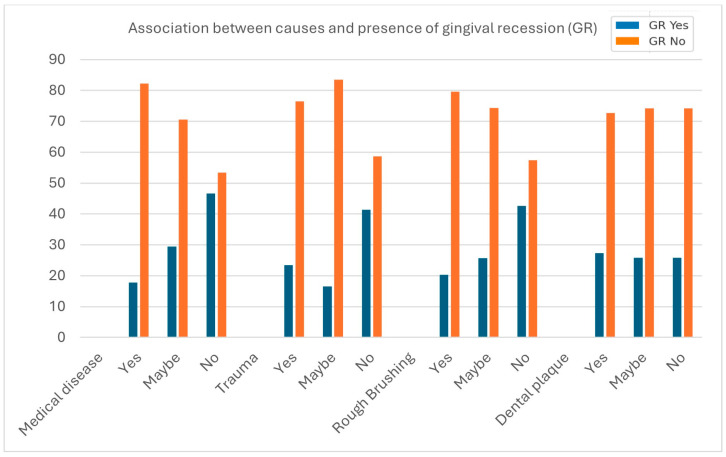
Association between causes and presence of gingival recession.

The analysis demonstrated significant associations between the presence of gingival recession (GR) and self-reported medical disease, trauma, and rough brushing habits (all *p* < 0.0001). Participants without medical disease, trauma, or rough brushing showed markedly higher prevalence of GR compared to those who acknowledged or suspected these factors, suggesting possible underreporting or misperception of causative factors among participants. In contrast, dental plaque did not show a significant association with GR (*p* = 0.9224), Figure 3. 

## 4. Discussion

In our study, the participants were mostly in early adulthood, between 20 and 39 years old, representing 66% of our total participants; 165 participants reported having GR out of 619, representing 26.5%. A previous study [14] conducted on 1000 participants, 56.70% reported that gingival recession is a sign of periodontal disease. Naser, M. Y. et al. [15] surveyed the Jordanian population, and more than half (59.0%) out of 1099 participants were aged 26 years and below. West, N. X. et al. [16] studied the prevalence of perception of gingival recession in seven European countries, with a mean age of 44 ± 17.4 years among more than 3500 participants.

In our study, female participants represented 48.47% of the sample compared to 51.5% males. This contrasts with higher female participation rates (55.1–63.9%) reported in other studies [13,14,15,16]. Notably, Saudi females generally demonstrate greater oral health awareness than males, with more frequent toothbrushing and dental visits [17]. However, despite better preventive practices, females were reported to exhibit higher gingival recession prevalence, potentially linked to brushing techniques or toothbrush selection [18]. Our regression analyses were adjusted for age, gender, socioeconomic status, and smoking, ensuring these factors did not confound the observed associations.

The higher prevalence of gingival recession among males and smokers observed in our study could be attributed to behavioral factors such as more aggressive brushing techniques, higher tobacco consumption, and less frequent use of preventive dental services compared to females. Age-related physiological changes, including cumulative mechanical wear of the gingiva and reduced tissue regenerative capacity, may also contribute to the increased prevalence observed in older participants. Furthermore, systemic conditions associated with aging, such as diabetes, may exacerbate periodontal vulnerability and recession risk.

The study revealed that more than half of the participants had attained a university-level education, and only 13.7% perceived being affected by gingival recession GR. Educational level has been suggested to correlate with oral health awareness and maintenance. Studies reported that higher education levels were associated with better oral hygiene and less periodontal complication [15,19]. Educated individuals are more likely to adopt preventive measures and seek regular dental care [20] while lower socioeconomic and educational status may increase GR risk due to limited access to preventive care [18,19,20,21]. However, Checchi et al. emphasized that while education plays a role in shaping oral hygiene practices, specific behaviors, such as aggressive brushing techniques or infrequent dental visits) can override its benefits [22]. These findings highlight the complex relationship between education and dental disease awareness and perception [23], highlighting the need for targeted interventions addressing both knowledge and behavior in vulnerable populations. Lower income and education levels were linked to poorer oral health indicators, suggesting that socioeconomic disparities significantly affect oral health outcomes [24,25]. People with higher socioeconomic often have better access to information and resources that promote oral health. This includes access to educational materials, preventive care, and professional dental services. In contrast, those with lower SES may lack these resources, leading to lower awareness and poorer oral health practices [26,27].

Our findings reveal low rates of regular dental attendance, while more than half of the participants reported seeking care only when experiencing symptoms. These patterns align with previous study [14], which reported similar trends of symptom-driven dental utilization.

Almost a third of the participants reported brushing twice and using a random brushing technique. Improper brushing techniques, and the use of medium or hard toothbrushes are closely linked to gingival recession. These practices can cause mechanical trauma to the gingiva, leading to recession [21,22,28]. Awareness of proper brushing techniques is crucial. Educating the population on the importance of using soft-bristled toothbrushes and adopting vertical or circular brushing methods can help reduce the incidence of gingival recession [23,29].

Although 40% of the participants perceived dental hypersensitivity, 70% of the participants did not report having GR. Dentin hypersensitivity (DH) is characterized by a sharp, short pain from exposed dentin in response to various stimuli, such as thermal or chemical triggers. It is often linked to the exposure of dentinal tubules due to enamel loss or gingival recession [30]. While gingival recession, the apical migration of the gingival margin can expose the root surface, leading to dentin exposure and potential hypersensitivity. However, not all cases of gingival recession result in hypersensitivity, and vice versa [31,32]. Despite the association between gingival recession and hypersensitivity, a study found that 79% of patients had gingival recession, but only 23.6% of these patients reported hypersensitivity, indicating that not all gingival recession cases lead to hypersensitivity [33].

The discrepancies between our findings and those reported in previous studies [15,16] may be attributed to methodological and demographic differences. While many international studies employed clinical examinations or combined methods, our reliance on self-reported questionnaires could have underestimated actual prevalence due to limited patient awareness or underreporting. Furthermore, our sample had a younger age distribution than populations assessed in European and Jordanian studies [33,34], which may partly explain the lower prevalence of self-reported gingival recession. Cultural factors, oral health behaviors, and differences in health literacy may also contribute to variations across studies. These discrepancies highlight the need for standardized approaches and cross-cultural validation of self-report measures to improve comparability.

The perception of hypersensitivity can be influenced by individual pain thresholds, oral hygiene practices, and awareness of dental conditions. Brushing habits and dietary choices were reported to exacerbate or mitigate symptoms [34]. A lack of awareness about the link between gingival recession and hypersensitivity may lead to underreporting. A study highlighted that many periodontitis patients were unaware of the connection between their symptoms and gingival recession [35].

Tooth sensitivity reports frequently encompass multiple etiologies beyond DH, including caries and periodontal diseases, potentially inflating prevalence estimates [34]. Current research methodologies fall into two primary categories: self-reported questionnaires and clinical examinations. However, both approaches yield wide prediction intervals (13–57% and 4–74%, respectively), reflecting substantial diagnostic variability. This dispersion may be attributed to diverse sample characteristics such as ethnic background, occupational environment, periodontal health, oral care practices, and socioeconomic factors [36,37].

DH diagnosis remains methodologically challenged with two principal paradigms emerging: a subjective approach based on patient-reported pain experiences versus an objective approach employing controlled thermal or mechanical stimuli [37,38,39]. Further complicating assessment is the condition’s intermittent nature, which may spontaneously exacerbate or relieve symptoms [37]. Dhaliwal et al. [39] reported a higher incidence of DH via questionnaire (48.9%). Nevertheless, compared with clinical methodology, he found only (25.0%) of the same Indian population.

The majority of our participants reported not knowing the treatment of hypersensitivity. West et al. clarify that dentin hypersensitivity was the condition for which participants most frequently used home treatments, such as toothpaste (58.7%). In comparison, the percentage of participants who had received professional treatment from their dentist for their dentin hypersensitivity was 54.9% [16].

Despite the prevalence of gingival recession, patient awareness of its causes and consequences is often limited. Many individuals associate the condition primarily with poor oral hygiene [15]. overlooking other contributing factors [40]. Gingival recession is often asymptomatic and not perceived by patients, which can limit the number of individuals seeking treatment [41]. Thus, educating patients about the multifactorial nature of gingival recession and the importance of proper oral hygiene and regular dental check-ups is crucial for prevention and management [40,42].

This emphasizes the importance of increasing patient awareness and education to promote early detection and intervention. In addition, emerging minimally invasive approaches such as injectable platelet-rich fibrin (i-PRF), platelet-rich plasma (PRP), and hyaluronic acid have been suggested as potential initial treatment options for gingival augmentation [43]. These biologically based modalities aimed to enhance soft tissue thickness and improve the healing environment without the morbidity associated with graft harvesting. Additionally, the focus on aesthetic concerns in treatment may overlook the broader implications of gingival recession, such as its impact on dental health and quality of life. Addressing these issues requires a comprehensive approach that considers both the aesthetic and functional aspects of dental care.

The medical conditions, traumatic factors, and brushing technique appeared to play a more prominent role in the development of GR than dental plaque in this studied cohort. Medical conditions like diabetes were acknowledged by a substantial portion of participants as contributing to recession, though this awareness varies across different populations [13,15]. Traumatic factors [13,15] and improper brushing techniques [16] were less commonly identified as potential causes, despite established clinical evidence. While many participants correctly associated plaque accumulation with recession, a notable proportion in a previous study [13] failed to recognize this relationship.

The connection between gingival recession and tooth mobility was widely recognized among respondents as being a consequence to periodontitis leading to potential tooth loss [13,15,16]. Clinical observations confirm that recession often precedes root surface exposure, leading to hypersensitivity and caries susceptibility. Global trends indicate worsening periodontal health outcomes in recent decades [16,44].

The current findings highlight the need for targeted preventive strategies in Saudi Arabia. Public health campaigns should focus on discouraging harmful habits such as smoking, promoting safe brushing techniques, and encouraging regular preventive dental visits rather than symptom-driven care. Dentists and hygienists can play a pivotal role by incorporating tailored patient education into routine check-ups, particularly for high-risk groups such as males, smokers, and older adults. School-based oral health programs and community outreach may further enhance awareness and promote healthier behaviors from an early age.

The findings of this research contribute to our understanding of public perceptions regarding gingival recession and its associated risk factors. A strength of this study is adjustment for major confounders, although residual confounding cannot be fully excluded. However, several limitations must be considered when interpreting these findings. First, the cross-sectional design prevents causal inference, and the reliance on self-reported data may have introduced recall bias. Participants may have misclassified or failed to recognize gingival recession or dentin hypersensitivity, leading to potential underreporting or misestimation of prevalence. Online and clinical recruitment leads to the risk of selection bias, since health-conscious individuals are more likely to participate in such studies. Thus, our results should be interpreted as perceptions and awareness levels rather than clinically verified diagnoses. Secondly, the use of convenience sampling limits the representativeness of the sample, potentially skewing results toward younger, more educated, or more health-aware individuals, which could underestimate true prevalence in the general Saudi population. Finally, the absence of clinical validation of self-reports likely reduced the accuracy of the findings; for example, participants with subclinical gingival recession may not have recognized or reported it. These limitations suggest that the true burden of gingival recession and hypersensitivity may be higher than observed in our study. Future studies would benefit from longitudinal designs, probability sampling, and combining clinical validation to strengthen the evidence base.

## 5. Conclusions

The perception of gingival recession remains limited among Saudi adults, despite its clinical and sensible significance. Awareness gaps were particularly evident regarding its association with hypersensitivity and caries. These findings show the urgent need for structured preventive programs, including community-based oral health campaigns and integration of gingival health education into broader public health initiatives. Dentists and dental hygienists play a central role by incorporating targeted education into routine dental visits, with emphasis on safe brushing techniques, early recognition of gingival changes, and timely referral for management. Personalized educational plans that consider socioeconomic and educational disparities are recommended to maximize outreach and impact.

## Figures and Tables

**Table 1:** Descriptive statistical analysis of the study findings.

Variable		*n* = 619	
	mean ± standard deviation (SD)	*n*	%
Age	30.88 ± 11.09 years		
20–29		245	39.58
30–39		164	26.49
40–49		137	22.13
50–59		62	10.02
>60		11	1.78
Gender			
Male		319	51.53
Female		300	48.47
Marital state			
Single		188	30.37
Married		416	67.21
Divorced		11	1.78
Widow		4	0.65
Education Level			
Elementary		19	3.07
Middle		39	6.30
High school		202	32.63
University		359	58.00
Socioeconomic status			
low < 7000 SR		349	56.38
Medium 7000–20,000		225	36.35
High > 20,000		45	7.27
Medical diseases			
Yes		272	43.9%
No		347	56.09%
Smoking habit			
Non		529	85.46
Light (1–10 cigarettes per day)		19	3.07
Medium (11–20 cigarettes per day)		43	6.95
Heavy (>20 cigarettes per day)		14	2.26
Former		14	2.26
Dental clinics			
Public		250	40.39
Private		369	59.61
Dental visits			
Regular		72	11.63
Irregular		160	25.85
with Pain		387	62.52
Brushing frequency			
3/day		50	8.08
2/day		201	32.47
1/day		197	31.83
Never		15	2.42
Irregular		156	25.20
Brushing technique			
Horizontally		127	20.52
Vertically		99	15.99
Circularly		184	29.73
Randomly		209	33.76
Number of missing teeth	1.37 ± 1.65		
0		288	46.53
1		110	17.77
2		71	11.47
3		43	6.95
4		67	10.82
>4		40	6.46
Do you have Hypersensitivity?			
Yes		252	40.71
No		367	59.29
Number of teeth affected by hypersensitivity	1.45 ± 1.83		
0		330	53.31
1		44	7.11
2		76	12.28
3		44	7.11
4		56	9.05
>4		69	11.15
Is there treatment for hypersensitivity?			
Yes		115	18.58
No		504	81.82
Do you have Gingival Recession?			
Yes		165	26.66
No		454	73.34
Number of teeth affected with gingival recession	0.89 ± 1.60		
0		439	70.92
1		31	5.01
2		44	7.11
3		28	4.52
4		35	5.65
>4		42	6.79

**Table 3:** Bivariate chi-square analysis of perceived consequences and presence of gingival recession (GR).

	Presence of GR		
Mobility	Yes	No	*p*
Leads to	26.52	73.48	0.9601
I do not know	27.15	72.85	
Irrelevant	25	75	
Teeth loss	Yes	No	*p*
Leads to	25.69	74.31	0.6367
I do not know	27.19	72.81	
Irrelevant	32.5	67.5	
Periodontitis	Yes	No	*p*
Leads to	27.31	72.69	0.8442
I do not know	25.3	74.7	
Irrelevant	23.81	76.19	
Caries	Yes	No	*p*
Leads to	32.1	67.9	0.0472 *
I do not know	23.46	76.54	
Irrelevant	22.41	77.59	

* *p* < 0.05 showing significant difference.

## Data Availability

All data are presented in the paper, further information will be provided upon request from the corresponding author.

## Supplementary Materials

The following supporting information can be downloaded at: https://www.mdpi.com/article/10.3390/dj13110501/s1.
