# Supplementary material for: Awareness of Gingival Recession and Its Causes and Consequences Among Adults in Saudi Arabia
Source: Dent J (Basel). 2025 Oct 28;13(11):501. doi: 10.3390/dj13110501 (PMC12650827; doi:10.3390/dj13110501)
Supplement: Supplementary file 1 [file dentistry-13-00501-s001.zip › dentistry-3869295-supplementary.pdf]

Supplementary Material S1

Awareness of Gingival Recession and Its Causes and Consequences Among Adults in Saudi Arabia

We kindly invite you to participate in this survey, which aims to assess awareness, perceptions, and contributing factors related to gingival recession among adults. Your responses will remain anonymous, and you withdraw at any point. Your responses will be used solely for research purposes.

The survey tool

1. What is your age?	20-29<br>30-39<br>40-49<br>50-59<br>> 60
2. What is your Gender?	Male<br>Female
3. What is your marital status?	Single<br>Married<br>Divorced<br>Widow
4. What is your educational level?	Elementary<br>Middle<br>High school<br>University
5. 6. What is your socioeconomic status?	low <7000 SR<br>Medium 7000-20000<br>High >20000
6. Do you have any medical diseases?	Yes<br>No
7. What is your smoking habit?	Non<br>Light (1-10 cigarettes per day)<br>Medium (11-20 cigarettes per day)<br>Heavy (>20 cigarettes per day)<br>Former
8. What type of Dental Clinics do you commonly visit?	Public<br>Private
9. How regular are your dental visits?	Regular<br>Irregular<br>with Pain
10. What is your teeth brushing frequency?	3/day<br>2/day<br>1/day<br>Never<br>Irregular
11. What is your tooth brushing technique?	Horizontally<br>Vertically<br>Circularly<br>Randomly
12. What is the number of missing teeth (except wisdom teeth)?	0<br>1

	2<br>3<br>4<br>>4
13. Do you have dentine hypersensitivity?	Yes<br>No
14. How many teeth are affected by dentine hypersensitivity?	0<br>1<br>2<br>3<br>4<br>>4
15. Do you know the treatments for dentine hypersensitivity?	Yes<br>No
16. Do you have gingival recession as you noticed?	Yes<br>No
17. How many teeth are affected by the gingival recession as you noticed?	0<br>1<br>2<br>3<br>4<br>>4
18. Do you think that **Medical diseases** are causes of gingival recession?	Yes<br>May be<br>No
19. Do you think that **Trauma** is cause of gingival recession?	Yes<br>May be<br>No
20. Do you think that **Rough brushing** is cause of gingival recession?	Yes<br>May be<br>No
21. Do you think that **Plaque accumulation** is cause of gingival recession?	Yes<br>May be<br>No
22. Do you know about the consequences of gingival recession (**esthetic appearance)?**	Leads to<br>I don't know<br>Irrelevant
23. Do you know about the consequences of gingival recession (**Tooth mobility)?**	Leads to<br>I don't know<br>Irrelevant
24. Do you know about the consequences of gingival recession (**Tooth loss**)?	Leads to<br>I don't know<br>Irrelevant

25. Do you know about the consequences of gingival recession **(Periodontitis**)?	Leads to<br>I don’t know<br>Irrelevant
26. Do you know about the consequences of gingival recession (**Caries)?**	Leads to<br>I don’t know<br>Irrelevant
27. Do you know the treatments for gingival recession?	Yes<br>No
